# Intensity-Duration-Frequency (IDF) rainfall curves, for data series and climate projection in African cities

**DOI:** 10.1186/2193-1801-3-133

**Published:** 2014-03-09

**Authors:** Francesco De Paola, Maurizio Giugni, Maria Elena Topa, Edoardo Bucchignani

**Affiliations:** DICEA, Università di Napoli Federico II, Napoli, Italy; AMRA S.c.a r.l, Via Nuova Agnano, Napoli, Italy; Centro Euro-Mediterraneo sui Cambiamenti Climatici (C.M.C.C.), Via Maiorise, Capua (CE), Italy; Centro Italiano Ricerche Aerospaziali (C.I.R.A.), Via Maiorise, Capua (CE), Italy

**Keywords:** Intensity, Duration, Frequency curves, Disaggregation analysis, Climate change, Africa

## Abstract

Changes in the hydrologic cycle due to increase in greenhouse gases cause variations in intensity, duration, and frequency of precipitation events. Quantifying the potential effects of climate change and adapting to them is one way to reduce urban vulnerability. Since rainfall characteristics are often used to design water structures, reviewing and updating rainfall characteristics (i.e., Intensity–Duration–Frequency (IDF) curves) for future climate scenarios is necessary (Reg Environ Change 13(1 Supplement):25-33, 2013).

The present study regards the evaluation of the IDF curves for three case studies: Addis Ababa (Ethiopia), Dar Es Salaam (Tanzania) and Douala (Cameroon). Starting from daily rainfall observed data, to define the IDF curves and the extreme values in a smaller time window (10′, 30′, 1 h, 3 h, 6 h, 12 h), disaggregation techniques of the collected data have been used, in order to generate a synthetic sequence of rainfall, with statistical properties similar to the recorded data. Then, the rainfall pattern of the three test cities was analyzed and IDF curves were evaluated.

In order to estimate the contingent influence of climate change on the IDF curves, the described procedure was applied to the climate (rainfall) simulations over the time period 2010–2050, provided by CMCC (Centro Euro-Mediterraneo sui Cambiamenti Climatici). The evaluation of the IDF curves allowed to frame the rainfall evolution of the three case studies, considering initially only historical data, then taking into account the climate projections, in order to verify the changes in rainfall patterns. The same set of data and projections was also used for evaluating the Probable Maximum Precipitation (PMP).

## Introduction

Degradation of water quality, property damage and potential loss of life due to flooding is caused by extreme rainfall events. Historic rainfall event statistics (in terms of intensity, duration, and return period) are used to design flood protection structures, and many other civil engineering structures involving hydrologic flows (McCuen 1998; Prodanovic and Simonovic 2007).

Any change in climate produces modifications in extreme weather events, such as heavy rainfall, heat and cold waves, in addition to prolonged drought occurrences (Almazroui et al. 2012).

Since rainfall characteristics are often used to design water structures, reviewing and updating rainfall characteristics (i.e., Intensity–Duration–Frequency (IDF) curves) for future climate scenarios is necessary (Mirhosseini et al. 2013).

A lot of studies, especially recently, have been developed to analyze the factors for assessment, adaptation and mitigation of climate change, and to enhance and sharpen the disaster management for the many and various stakeholders.

El-Hadji and Singh (2002), using long-term data (from 1951 to 1990) of rainfall and annual runoff, have developed an investigation of spatial and temporal variability of rainfall and runoff in the Casamance River basin, located in southern Senegal, West Africa.

Chowdhury and Beecham (2010,2012) have analyzed the effects of changing rainfall patterns in Australia, in order to define the design parameters of Water Sensitive Urban Design (WSUD) technologies, as bioretention basins and permeable pavements, describing how these systems behave under varying rainfall conditions.

Kuhn et al. (2011) have analyzed the effect of climate change and continuing land use change in the Gallocanta Basin (Spain), one of only a few bird sanctuaries. Therefore, in order to obtain an appropriate management of the bird sanctuary, it was important to understand the impact of climate change on basin hydrology in terms not only of total amount of rainfall, but also considering the individual extreme events, that affect the basin level.

Sherif et al. (2011,2013) have analyzed spatial and temporal characteristics of rainfall in the United Arab Emirates (UAE). The rainfall patterns, rainfall probability of occurrence, rainfall intensity-duration-frequency (IDF) relationship, probable maximum precipitation (PMP) and drought scenarios were investigated, using standard statistical techniques, as Gumbel, log Pearson, GEV, log normal, Wakeby and Weibull probability distributions.

The IDF curves were also estimated for 13 stations in Cote D’Ivoire in the Sora et al. study, using rainfall data series of durations ranging from 15 minutes to 4 hours.

**Figure 1:**
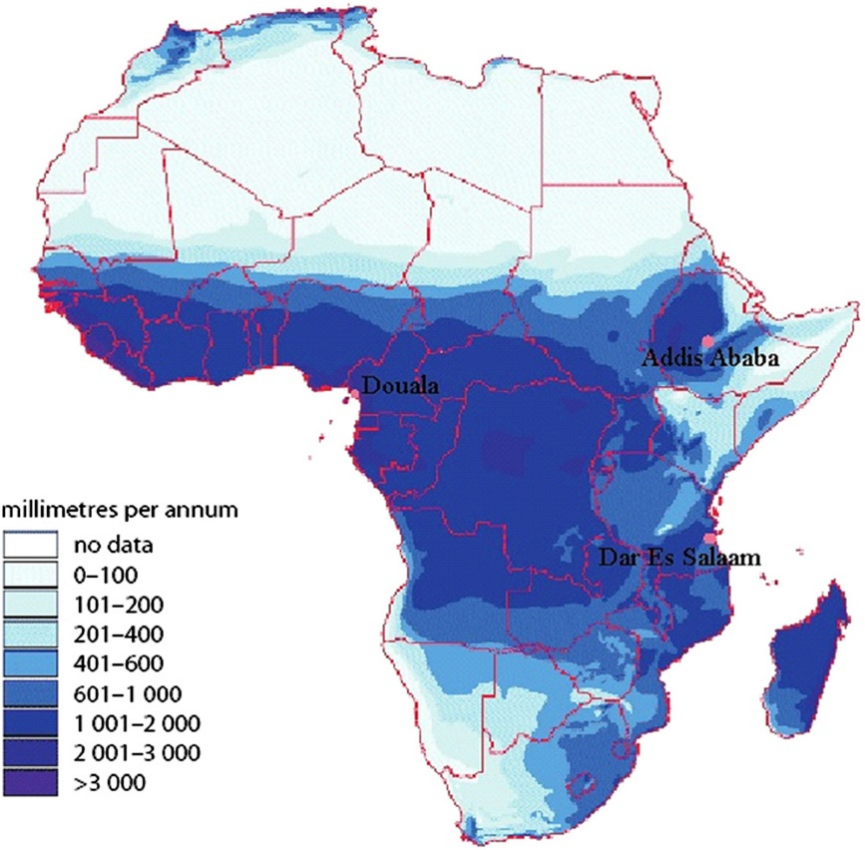
**Map of rainfall variability in Africa [FAO/Agrhymet Network and ESRI].**

The estimation and use of IDF curves, as shown also in some of the cited works, rely on the hypothesis of rainfall series stationarity, namely that intensities and frequencies of extreme hydrological events remain unchanged over time. In the present work, in order to assess how extreme rainfalls will be modified in a future climate, analysis of observed data and future simulations has been performed in three african test cities: Addis Ababa (Ethiopia), Dar Es Salaam (Tanzania) and Douala (Cameroon), characterized by different rainfall patterns (Figure 1).

Furthermore, in the three cities considered, available rainfall records are limited to daily time steps. Since rainfall data at shorter time steps are essential for the evaluation of IDF curves, a daily rainfall disaggregation model was adopted.

The climate projections used, provided by CMCC, were performed following the IPCC (Intergovernmental Panel on Climate Change) 20C3M protocol for the 20th Century, and the RCP4.5 and RCP8.5 radiative forcing scenarios for the 21st century: they are characterized by horizontal resolutions of about 8 km and 1 km.

In addition, for the three case studies, was also estimated, both for data series and projections, Probable Maximum Precipitation (PMP), for duration rating from 10′ to 24 h. Extreme rainfall features and estimates of PMP for different return periods, evaluated in this study, will be useful to planning and designing flood protection structures.

### Probability distribution for IDF

The intensity-duration-frequency curves are used in hydrology to express in a synthetic way, fixed a *return period T* and a *duration d* of a rainfall event, and for a given location, the information on the maximum rainfall height h and the maximum rainfall intensity i. Known these parameters, it is possible to build synthetic rain graphs that are useful to the elaboration of flood hydrographs.

Generally, IDF curves can be characterized by the expression:
1

in which a(T) and n are the parameters that have to be estimated through a probabilistic approach.

The *cumulative probability function P(h)* represents the probability of not exceeding the value of the rainfall height h by that random variable. In this case, the cumulative distribution function (CDF) used was the classical distribution of Gumbel (Maximum Extreme Value Type 1):
2

in which the parameters u and v are linked to the mean value (μ) and to the standard deviation (σ) through the following equations:
34

The inverse of CDF can be calculated by evaluating h in terms of P(h) and duration d:
5

Substituting the u and v expressions as functions of μ and σ, and introducing the variation coefficient CV, equal to σ/μ, is easy to obtain:
6

Since the probability P is related to the return period T by the simple relationship:
7

h can be expressed as a function of the return period T as:
8

where K is equal to:
9

Assuming that *μ*(*d*) = *a*_*μ*_*d*^*n*^, in which a_μ_ is the value of a related to the mean value μ(d), it is therefore obtained:
10

where CV_m_ is the mean CV value over different durations d. Taking into account the general expression (1), it follows that:
11

Assuming that *K*_*T*_ = (1 + *CV*_*m*_*k*_*T*_), generally called *growing facto*r, it is easy to obtain:
12

For a variation coefficient slightly variable with the duration d, the mean value CV_m_ can be evaluated by the following expression:
13

in which k is the considered duration, in this work equal to 7 (10′, 30′ minutes, 1, 3, 6, 12, and 24 hours).

### Disaggregation of daily rainfall data

The data available for the three test cities, provided by the links http://www.tutiempo.net and http://www.climexp.knmi.nl, concern only the maximum daily data for a specified year of observation.

In Table 1, the coordinates of the stations and the available data range for the three test cities are shown.

**Table 1:** **Characteristics of the meteorological stations of the three test cities**

	Longitude	Latitude	Altitude [m]	Available data range
**Addis Ababa**	38.8	9.03	2355	1964-2010
**Dar Es Salaam**	39.2	−6.86	55	1958-2010
**Douala**	9.73	4	10	1976-2010

In order to define the extreme values in a smaller time window (10′, 30′, 1 h, 3 h, 6 h, 12 h), a synthetic sequence of rainfall was generated, with statistical properties equal to those of the observed rainfall. In greater detail, the daily rainfalls have been successively disaggregated using two models:

-  cascade-based disaggregation model
-  short-time intensity disaggregation method

Assuming that daily rainfalls derive from a marked Poisson process, i.e. rainfall lag and heights are drawn from exponential probability density functions (whose parameters are calculated from observed rainfall series), it is possible to use a simple stochastic model of daily rainfall, that describes the occurrence of rainfall as a compound Poisson process with frequency of events λ. The distribution of times τ between precipitation events is an exponential with mean 1/λ, and exponentially distributed rainfall amounts h with mean γ. This model fits the observed daily data for individual seasons quite well.

**Figure 2:**
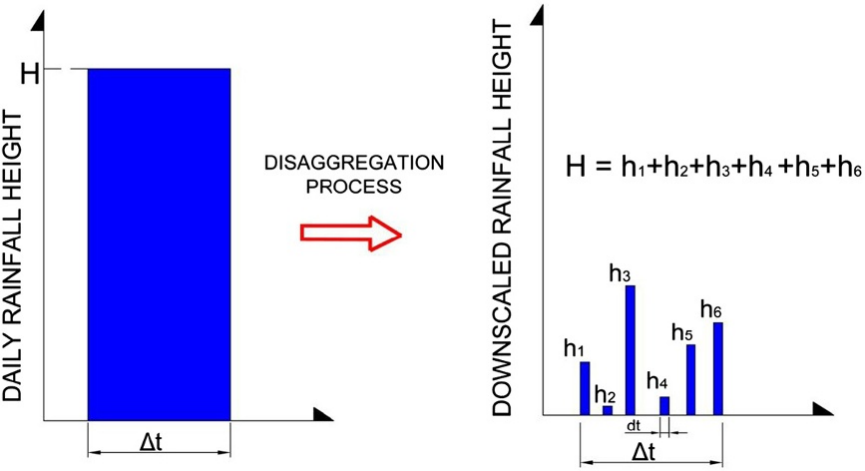
**Rainfall cascade disaggregation model applied: example of downscaled data.**

In a cascade-based disaggregation model (Güntner et al. 2001), daily precipitation data are converted into either 12-hourly, 6-hourly, or 3-hourly values, based on the principles of multiplicative cascade processes. For each year, known γ, λ, it’s possible to generate some years of disaggregated values, extracting the maximum value for each time window (3 h, 6 h, 12 h) (Figure 2).

Cascade level refers to the time series at a certain resolution. The transition from one cascade level to the higher one, corresponding to a doubling of resolution, is called *modulation*. A time interval at an arbitrary cascade level (i.e. time scale) is termed a *box*, which is characterized by an associated precipitation amount (0 if dry, >0 if wet). The break-up of a wet box into two equally sized sub-boxes is denoted *branching*. In one branching, the total amount is redistributed according to two multiplicative weights, 0 ≤ W_1_ ≤ 1 and 0 ≤ W_2_ ≤ 1 (W_1_ + W_2_ = 1). The model is a multiplicative random cascade of branching number 2 with exact conservation of mass (microcanonical property as opposed to canonical cascades where the volume is only approximately conserved). The model divides daily precipitation into non overlapping time intervals. If the precipitation in a day is P_d_, P_1_ = P_d_W_1_ is the precipitation amount assigned to the first half of the day, and P_2_ = P_d_W_2_ the amount assigned to the second half. Similarly, each half is then branched to a doubled resolution, and so on. The implementation of cascade – based model allows the conversion of daily amount into 12-hourly (1 steps), 6-hourly (2 steps), and 3-hourly (3 steps) values.

The short-time intensity disaggregation model (Connolly et al. 1998), is used to have three fine-resolution time interval, that are 1-hour, 1/2-hour and 10-minutes. A single Poisson distribution parameter represents the number of events, N, on a rainy day. The density function of the Poisson distribution (adjusted so that N > =1) has the form:
14

where η is a fitted coefficient. Mean (μ_N_) and variance (σ^2^_N_) are given as:
1516

The simulated number of event N is the lowest integer to satisfy:
17

where U is a uniform random number in the range 0–1.

The duration of each event, D, is represented with a gamma distribution. The scale parameter of the gamma distribution, α, has to be estimated and the shape parameter, β, is set held at 2. It results the following density function:
18

A uniform random number in the range 0–1, U, is generated and the event duration is simulated by solving the cumulative density function of the gamma distribution using Newton’s method:
19

To apply this model with reference to the case studies, the software CRA.clima.rain was used (Agricoltural Research Council - CLIMA version 0.3 2009).

With these estimated point (10′-30′-1 h, 3 h, 6 h, 12 h and 24 h) using the procedure described for the Gumbel distribution, it was possible to define the rainfall probability curves for the case studies.

### Estimation of the probable maximum precipitation: statistical approach

Probable Maximum Precipitation (PMP) is defined as the greatest depth of precipitation for a given duration meteorologically possible for a design watershed or a given storm area at a particular location at a particular time of year, with no allowance made for long-term climatic trends (World Meteorological Organization 1986).

The methodology used for estimating the PMP, is based on the Hershfield technique, founded on Chow (1951) general frequency equation:
20

and
21

where: X_M_,  and σ_n_ are the highest, mean and standard deviation for a series of n annual maximum rainfall values of a given duration;  and σ_n−1_ are the mean and standard deviation, respectively, for this series excluding the highest value from the series; and k_m_ is a frequency factor.

To evaluate this factor, initially Hershfield (1961) analysed 2645 stations (90% in the USA) and found an observed maximum value of 15 for k_m_, and so he recommended this value to estimate the PMP by equation (20). Later, Hershfield (1965) found that the value 15 was too high for rainy areas and too low for arid areas. Furthermore, it was too high for rain durations shorter than 24 h, so he constructed an empirical nomograph (World Meteorological Organization 1986) with k_m_ varying between 5 and 20 depending on the rainfall duration and the mean . Koutsoyiannis (1999) fitted a generalized extreme value (GEV) distribution to the frequency factors obtained from the 2645 stations used by Hershfield and found that the highest value 15 corresponds to a 60000-year return period, at the low end of the range considered by the NRC (National Research Council 1994). Casas et al. (2010) for the city of Barcelona assigned a frequency factor of 9.4 to this mean value: higher than any of the observed frequency factors, which vary from 2.1 to 6.6. In the present work, the value of k_m_, for different durations, was evaluated using the equation (21) and then also using the WMO nonmograph, as shown in the following paragraph.

### Climate change projection

The climate simulation has been performed following the IPCC (Intergovernmental Panel on Climate Change) 20C3M protocol for the 20th Century. The initial conditions were obtained from an equilibrium state reached by integrating the model for 200 years with constant greenhouse gases (GHGs) concentrations corresponding to 1950s conditions. Once the climate of the model was in equilibrium with the prescribed constant radiative forcing (GHG and aerosol concentrations), the simulations have been developed by increasing the GHG and aerosol concentrations in line with observed data.

The projections were performed using the RCP4.5 and the RCP8.5 emission scenarios, developed in the framework of the 5th Coupled Model Intercomparison project (CMIP5, http://cmippcmdi.llnl.gov/cmip5/).

CMCC has performed a set of climate simulations with the coupled global model CMCC-MED (resolution 80 km), over the time period 1950–2050. These simulations have been downscaled to a spatial resolution of about 8 km, performing regional simulations on three limited domains, including the cities of interest, with the regional model COSMO-CLM,

The CLM is the climate version of the COSMO model, which is the operational non-hydrostatic mesoscale weather forecast model developed by the German Weather Service. Successively, the model has been updated by the CLM-Community, in order to develop also climatic applications.

The output of climate models are affected by a systematic error, so the rainfalls projection outputs cannot be used in hydrological models or in decision making without performing some form of bias correction (Sharma et al. 2007; Hansen et al. 2006; Feddersen and Andersen 2005). A realistic presentation of future precipitation from climate models is extremely important for vulnerability and impact assessment (Wood et al. 2004; Schneider et al. 2007). Therefore, modelers use bias correction techniques to obtain more realistic outputs.

The bias correction technique adopted in this work is the “quantile mapping” one: mean and variability of the simulated values are corrected using the anomaly of the modeled cumulative frequency distribution compared to the observed cumulative frequency distribution. The algorithm systematically removes the median differences to zero and adopts the model output variance characteristics equal to the observed one.

A simulated value is the input to the process and is associated with a particular quantile in the simulated distribution. This same percentile is extracted from observed distribution and this quantile in the observed distribution becomes the bias corrected value.

Moreover, rainfall prediction on scales of order of a few kilometers in space and less than a hour in time is a necessary ingredient to issue reliable flood alerts in small area.

Downscaling techniques aim at generating an ensemble of stochastic realizations of the small-scale precipitation fields that have statistical properties similar to those measured for rainfall in a given area and/or synoptic situation. Since a downscaled precipitation field is the product of a stochastic process, it cannot be taken as a faithful deterministic prediction of small scale precipitation, but rather as one realization of a process with the appropriate statistical properties (Rebora et al. 2005).

The method introduced for stochastic rainfall downscaling is the Rainfall Filtered Autoregressive Model (RainFARM) and is based on the nonlinear transformation of a Gaussian random field: it conserves the information present in the rainfall fields at larger scales. By using this method it was possible to obtain, starting from the output of the regional model COSMO-CLM, climate projection at spatial resolution of about 1 km for the three case studies.

### Overview of the three case studies

#### *Addis Ababa*– *Ethiopia*

**Figure 3:**
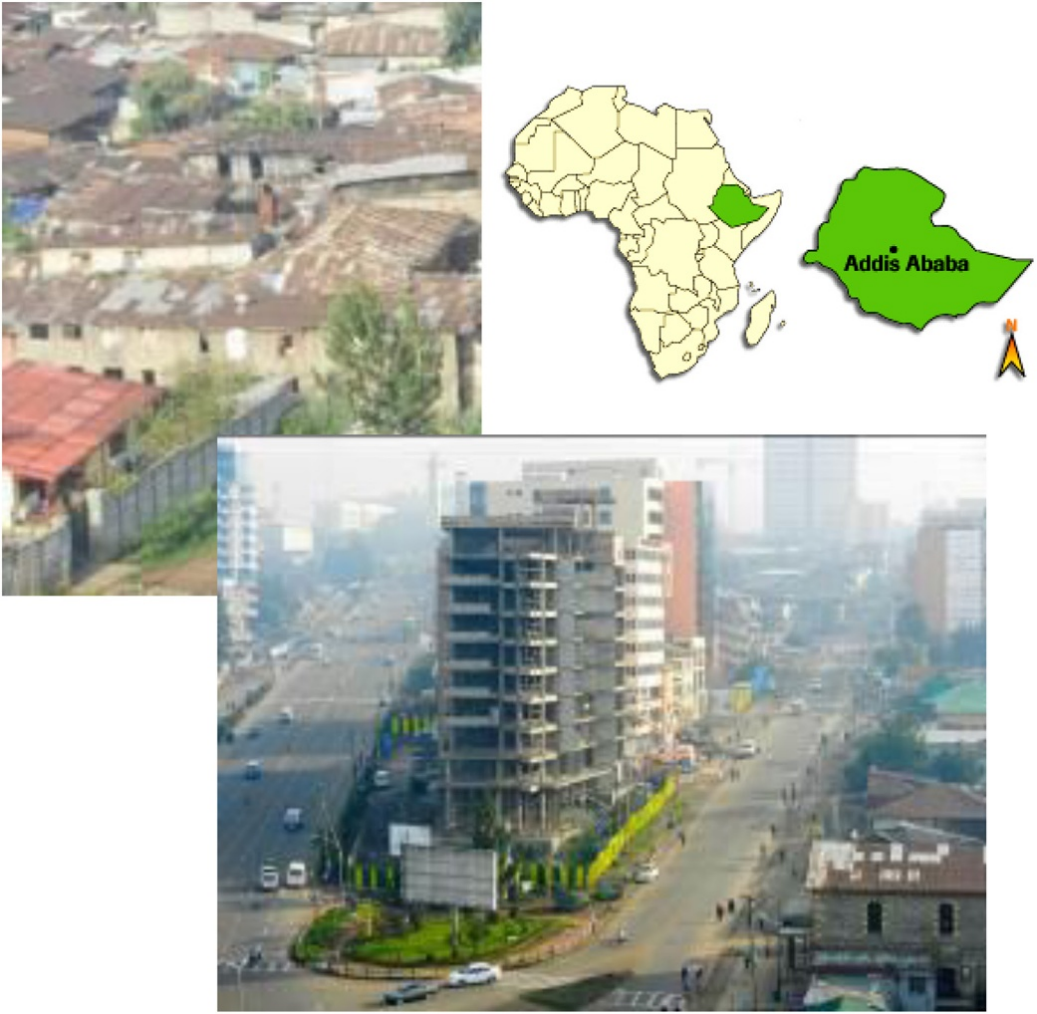
**The city of Addis Ababa (Giugni et al.** 2012 **).**

Addis Ababa (Figure 3) is the capital and the largest city of Ethiopia with 2,740,000 inhabitants based on the 2007 Census conducted by the Central Statistical Agency of Ethiopia (CSA), approximately 4 million based on the estimation of the UN-HABITAT Urban Profile.

The city is situated in the high plateau of central Ethiopia in the North–south oriented mountain systems neighboring the Rift-Valley.

The city is overlooked by mount Yarer to the east having approximately the same height as mount Entoto and mount Wochecha to the west, which is approximately 3361 m above sea level. The meteorological station, located in the relatively low altitude parts of the city, around Bole International airport, is at 2408 masl, while the elevation in Entoto mountain, north of the city, is more than 2444 masl.

Addis Ababa has a pronounced rainfall peak during the boreal summer (July to September) and exhibits a rainfall minimum during the boreal winter (November to February). The city has a temperate climate due to its high-altitude location in the subtropics. Mean annual precipitation vary between 730 mm, considering historical data, and 980 mm, considering climate projections (Giugni et al. 2012).

**Figure 4:**
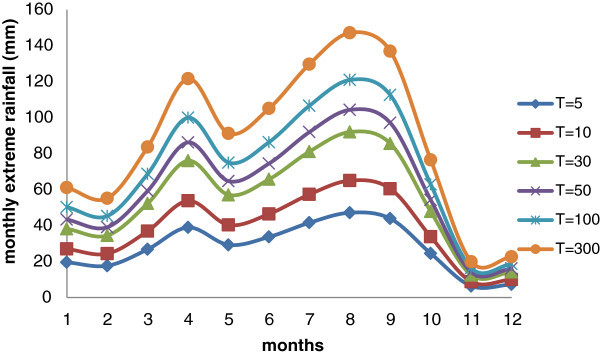
**Variation of monthly extreme rainfall for different return period for Addis Ababa (using historical data and climate projections – scenario RCP 8.5).**

The distribution of monthly maximum rainfall was also evaluated, using the distribution of Gumbel. Therefore the mean value, the standard deviation and the coefficient of variation, shown in Table 2, have been evaluated considering both the historical data and the climate projections (scenario RCP 8.5), including the assessment of the variation as a function of the return period. Figure 4 shows the monthly variation of the rainfall extremes for different values of return period, highlighting that the months of April and August are those in which the most extreme values have been evident.

**Table 2:** **Mean value μ, standard deviation σ and coefficient of variation c**_**v**_ **for the monthly maximum rainfall, evaluated in the different months, for the city of Addis Ababa**

	AddIs Ababa - 1964-2050											
	1	2	3	4	5	6	7	8	9	10	11	12
**μ**	10.48	9.45	14.33	20.84	15.63	18.00	22.23	25.21	23.46	13.11	3.42	3.89
**σ**	15.23	13.27	12.39	14.49	11.58	10.44	11.95	28.91	47.23	11.70	7.50	7.85
**c**_**v**_	1.45	1.40	0.86	0.70	0.74	0.58	0.54	1.15	2.01	0.89	2.19	2.02

#### *Dar Es Salaam*– *Tanzania*

**Figure 5:**
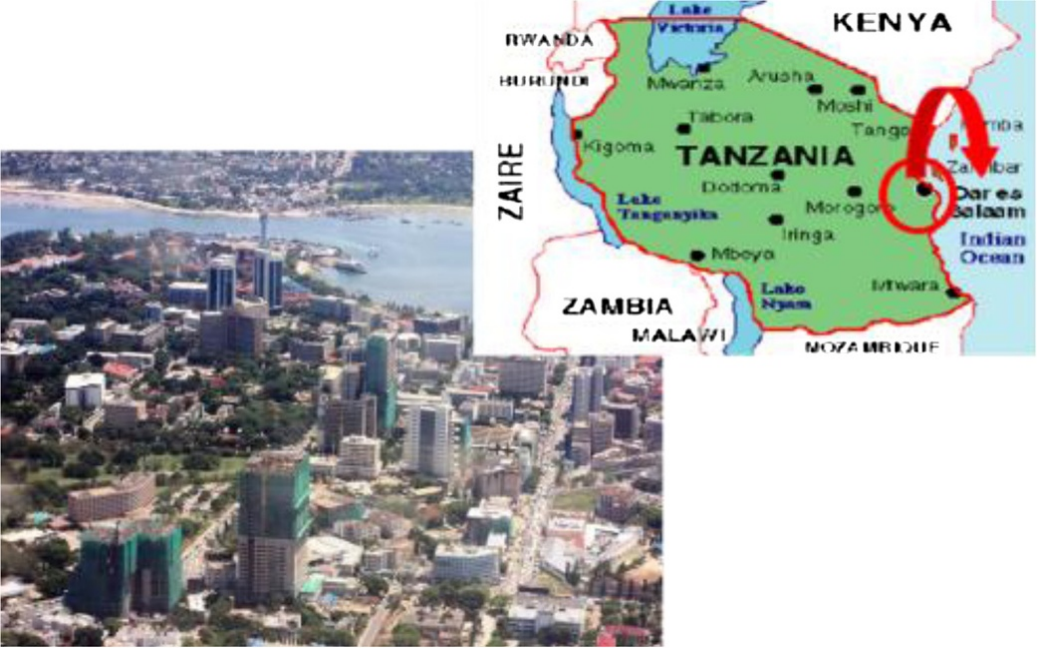
**The city of Dar Es Salaam (Giugni et al.** 2012 **).**

The City of Dar Es Salaam in Tanzania (Figure 5) is located between latitudes 6.36 degrees and 7.0 degrees to the south of Equator and longitudes 39.0 and 33.33 to the east of Greenwich. It borders Indian Ocean on the east and its coastline stretches about 100 km between the Mpiji River to the north and beyond the Mzinga River in the south.

Dar Es Salaam is the largest city in Tanzania with an estimated population of 3.4 million inhabitants.

The present day climate of Dar Es Salaam is characterized by a strong seasonal rainfall cycle, with the “long rains” from March to May, and the “short rains” from November to January, and a dry period from June to August. The mean annual rainfall is around 1000 mm.

**Figure 6:**
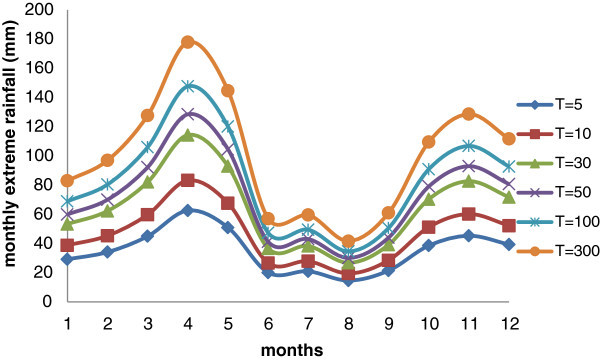
**Variation of monthly extreme rainfall for different return period for Dar Es Salaam (using historical data and climate projections – scenario RCP 8.5).**

As for the city of Addis Ababa, the distribution of monthly maximum rainfall was evaluated and the parameters of Gumbel distribution, mean value, standard deviation and coefficient of variation have been reported in Table 3. In Figure 6, the distribution of monthly maximum rainfall, considering different return period, was shown. For Dar Es Salaam, the extreme values are evident in April, indeed the months from June to September are the driest one.

**Table 3:** **Mean value μ, standard deviation σ and coefficient of variation c**_**v**_ **for the monthly maximum rainfall, evaluated in the different months, for the city of Dar Es Salam**

	Dar Es Salaam - 1958-2050											
	1	2	3	4	5	6	7	8	9	10	11	12
**μ**	17.44	20.37	26.84	37.37	30.39	11.98	12.53	8.76	12.82	23.01	27.04	23.47
**σ**	20.56	22.43	18.65	23.05	26.05	12.61	11.67	8.41	13.50	20.46	25.43	23.93
**c**_**v**_	1.18	1.10	0.69	0.62	0.86	1.05	0.93	0.96	1.05	0.89	0.94	1.02

#### *Douala*– *Cameroon*

**Figure 7:**
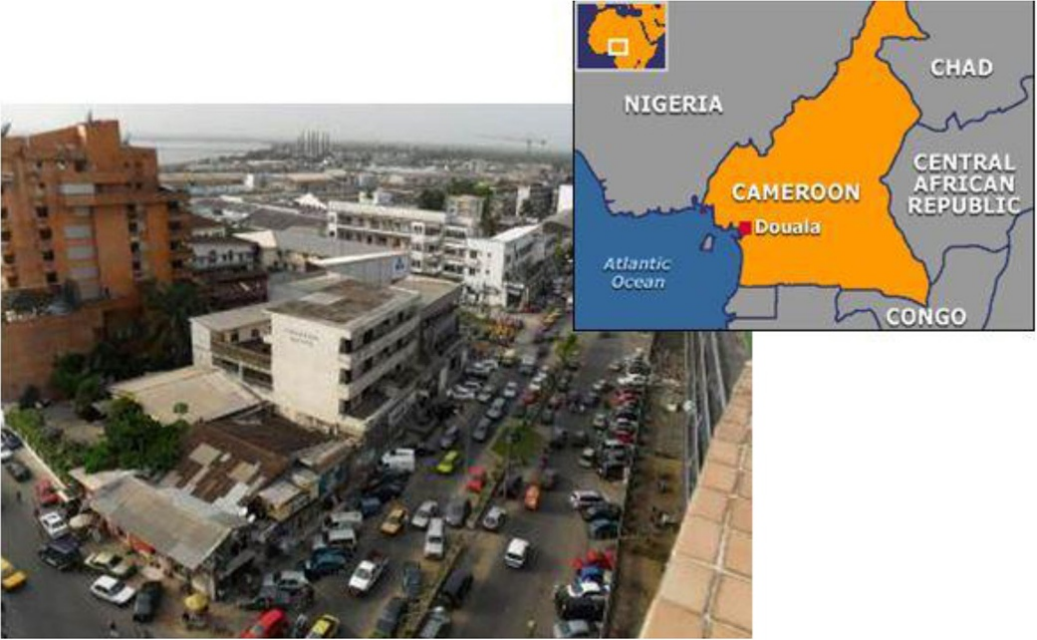
**The city of Douala (Giugni et al.** 2012 **).**

Douala (Figure 7) is the economic capital and the largest city of Cameroon with a population of about 2.1 million people (20% of Cameroon’s urban population, 11% of the country’s population) and an annual growth rate of 5% compared to the national average of 2.3%.

Douala experiences a wet, tropical monsoonal climate, with the average total annual rainfall exceeding 3000 mm.

Also for Douala, the distribution of monthly maximum rainfall was evaluated, using Gumbel distribution, and the parameters have been reported in Table 4.

**Table 4:** **Mean value μ, standard deviation σ and coefficient of variation c**_**v**_ **for the monthly maximum rainfall, evaluated in the different months, for the city of Douala**

	Douala - 1958-2050											
	1	2	3	4	5	6	7	8	9	10	11	12
**μ**	30.67	31.24	78.49	51.36	67.91	44.87	48.05	105.95	63.99	63.11	36.66	32.76
**σ**	39.26	40.75	259.63	62.71	240.34	34.65	31.60	328.92	45.22	60.47	33.48	35.34
**cv**	1.28	1.30	3.31	1.22	3.54	0.77	0.66	3.10	0.71	0.96	0.91	1.08

**Figure 8:**
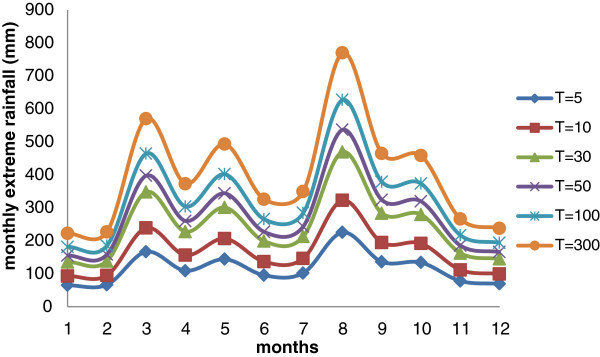
**Variation of monthly extreme rainfall for different return period for Douala (using historical data and climate projections – scenario RCP 8.5).**

Figure 8 illustrate this distribution, considering different return period, and shows that the maximum rainfall values occur in August.

Finally, it can be summarized that the three test cities are very differently located in position and altitude and are characterized by different rainfall patterns.

The city of Addis Ababa, with about 800 mm of mean annual rainfall, has a dry season during the months of November and December, while the highest rainfall values were recorded in the month of August. Dar Es Salaam, characterized by values of annual rainfall of about 1000 mm, shows maximum values in April.

At the end, Douala, with an average annual rainfall exceeding 3000 mm, has obviously the highest values of maximum rainfall with a peak in August.

### IDF curves, PMP and climate change

The procedure applied for the evaluation of the IDF curves was shown in the previous paragraphs. More in details, for each case study, the available daily rainfall data, for all years of observations, were disaggregated in 7 durations. In particular, using the cascade - based model, the values for the 3, 6 and 12 hours were obtained. In order to evaluate also durations less than three hours (10, 30 minutes and 1 hour), the short time intensity disaggregation model was used.

Durations less than an hour were chosen as they may cause flash flood events that often harm African cities (Murray and Ebi 2012; Douglas et al. 2008).

Once obtained the maximum values for the seven durations considered (10′, 30′ 1, 3, 6, 12 and 24 hours), these values were fitted by the Gumbel distribution and the IDF curves were evaluated, expressed in the form *μ*(*d*) = *a*_*μ*_*d*^*n*^.

The K_T_ values for different return periods were evaluated, in particular for 5, 10, 30, 100 and 300 years.

**Figure 9:**
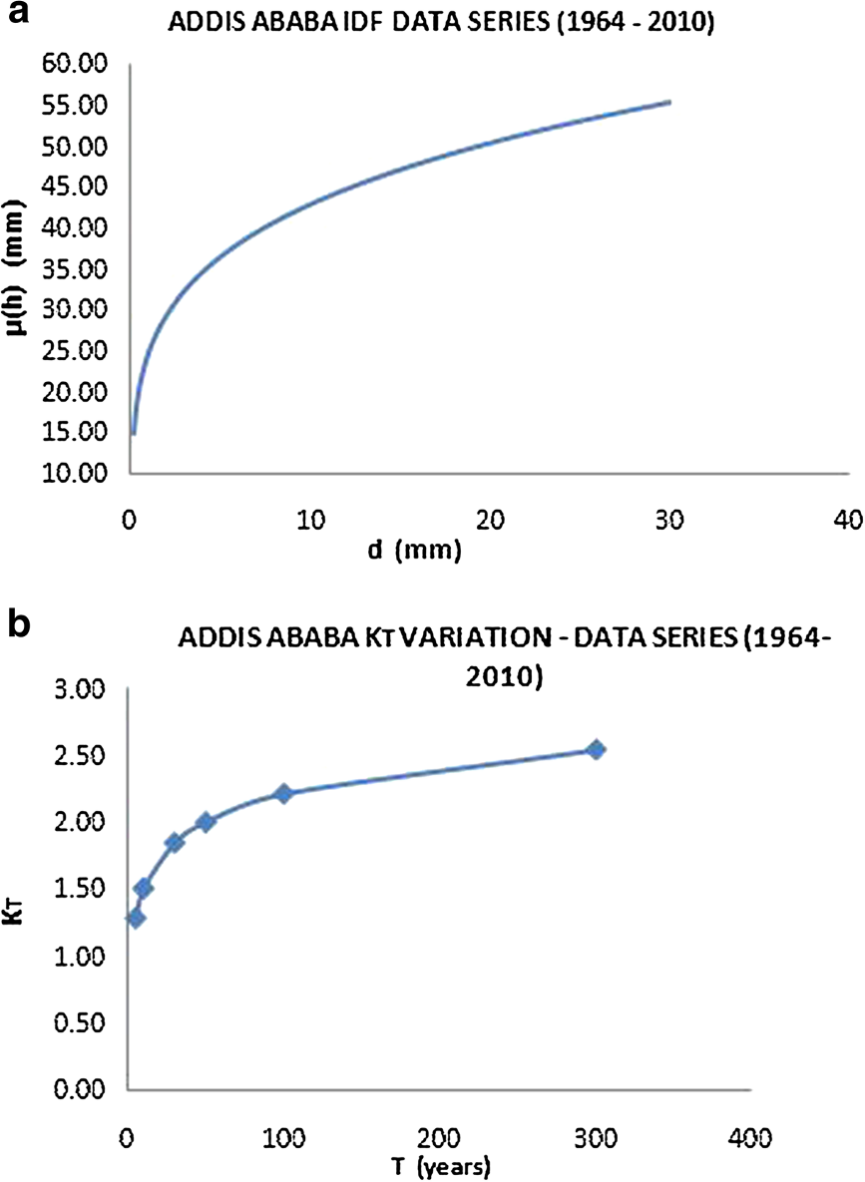
**IDF curve for the city of Addis Ababa (a) and variation of growing factor K**_**T**_ **(b).**

**Figure 10:**
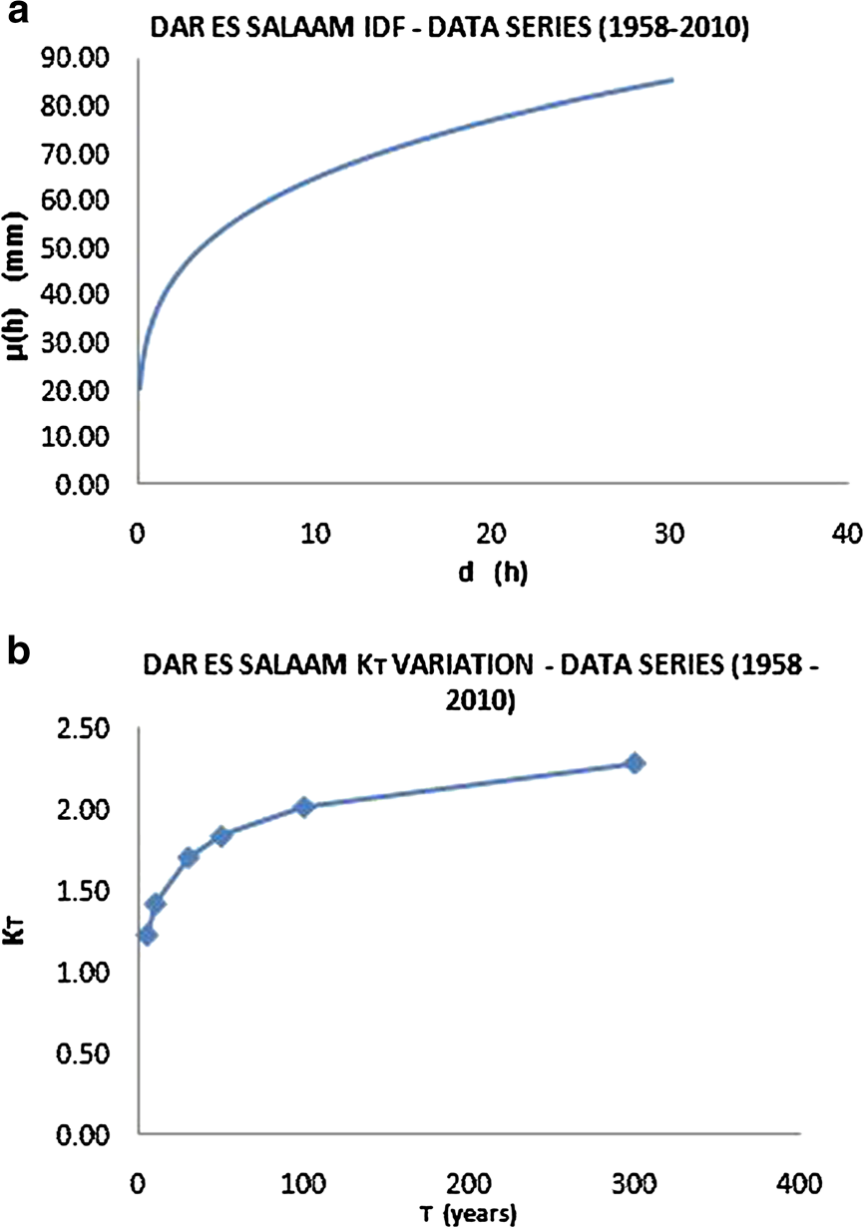
**IDF curve for the city of Dar Es Salaam (a) and variation of growing factor K**_**T**_ **(b).**

**Figure 11:**
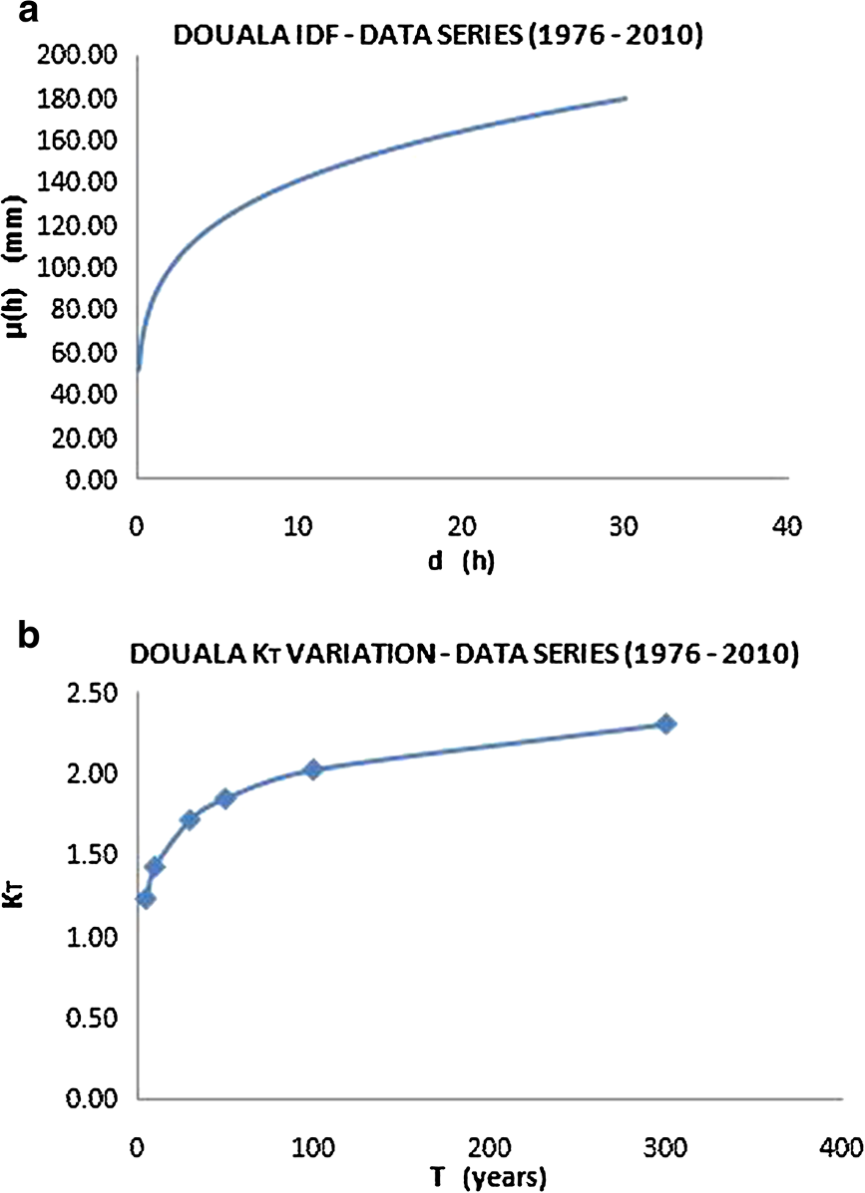
**IDF curve for the city of Douala (a) and variation of growing factor K**_**T**_ **(b).**

Initially, this procedure was applied only for the historical data series and the obtained results for the three test cities were shown in the Figures 9, 10 and 11.

The illustrated procedure was at a later stage applied to the climate simulations over the time period 2010–2050 provided by CMCC. More in details, the IDF curves for each test city were evaluated considering a rainfall series that consist of historical data and climate projections. In particular, the two emission scenarios RCP4.5 and RCP8.5 and the two different spatial resolutions, 8 km and 1 km, were considered, taking into account therefore four different options.

**Figure 12:**
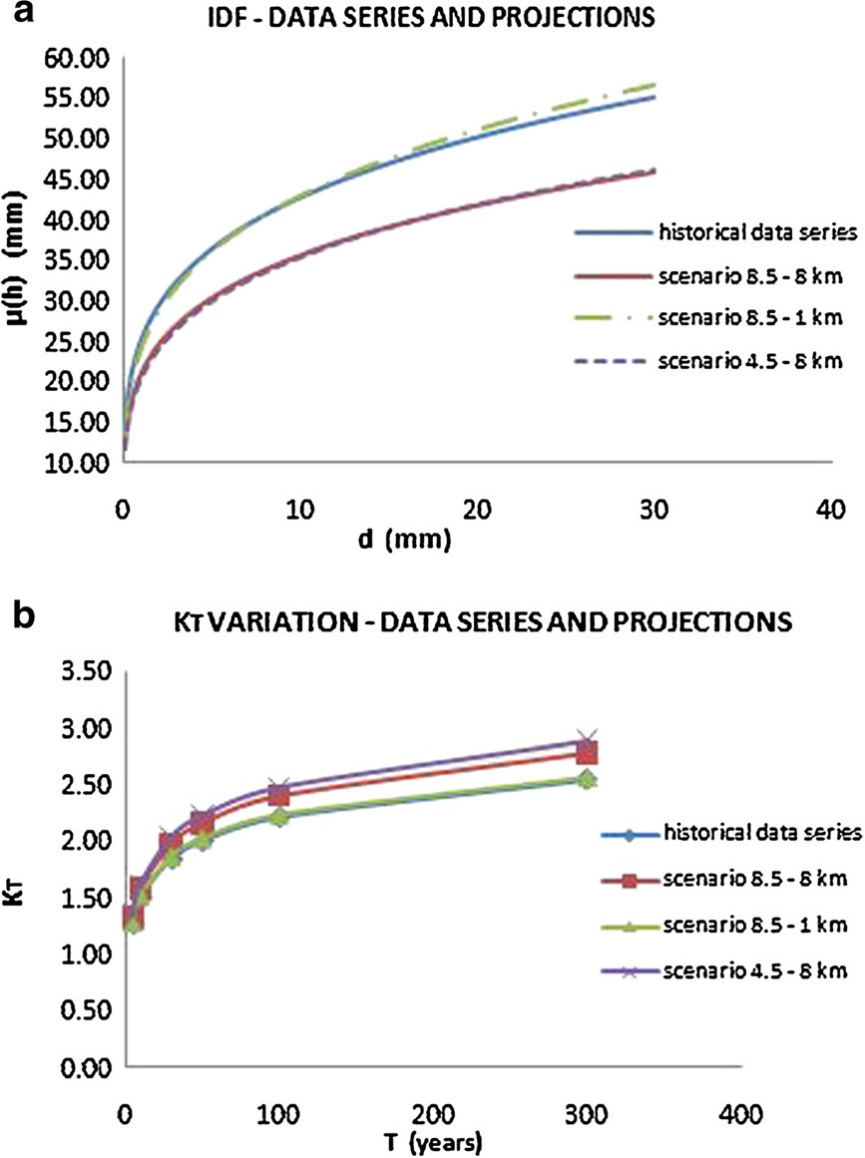
**IDF curves for the city of Addis Ababa (a) and variation of growing factor K**_**T**_ **(b) considering both historical data series and climate projections.**

**Figure 13:**
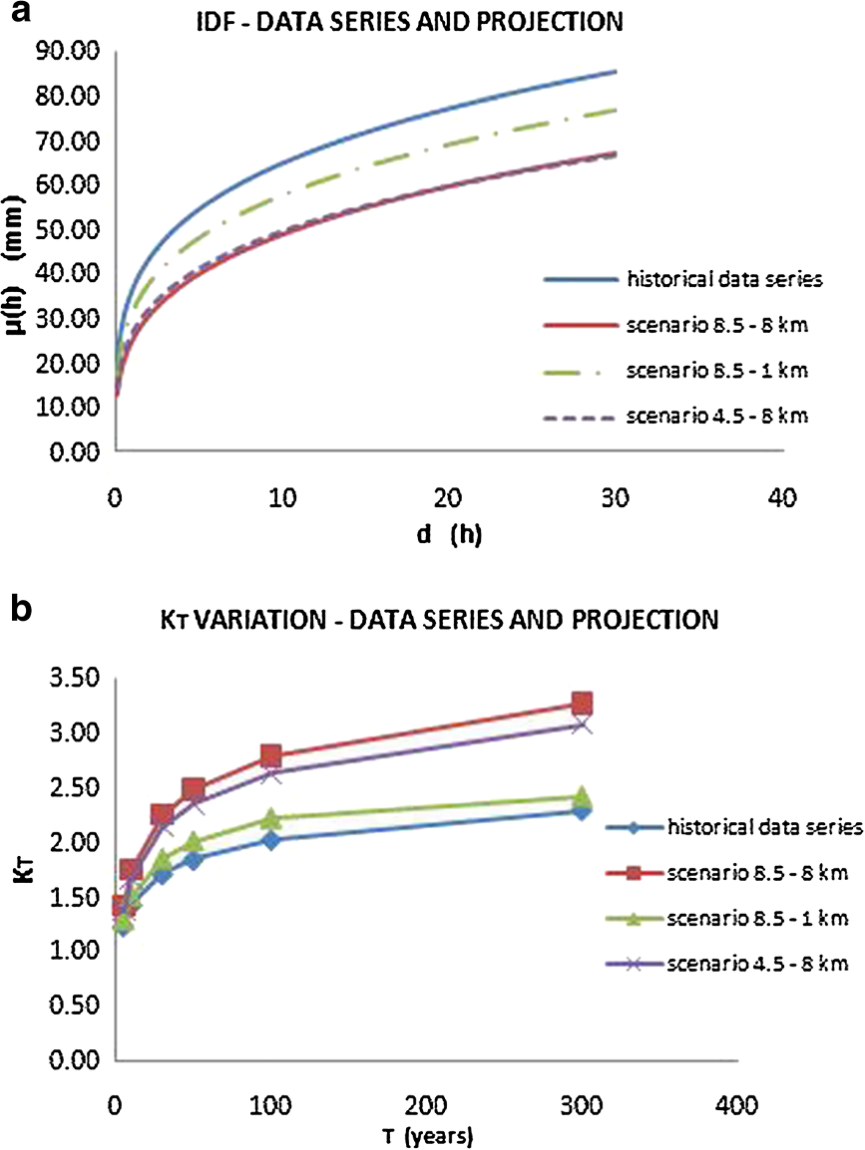
**IDF curves for the city of Dar Es Salaam (a) and variation of growing factor K**_**T**_ **(b) considering both historical data series and climate projections.**

**Figure 14:**
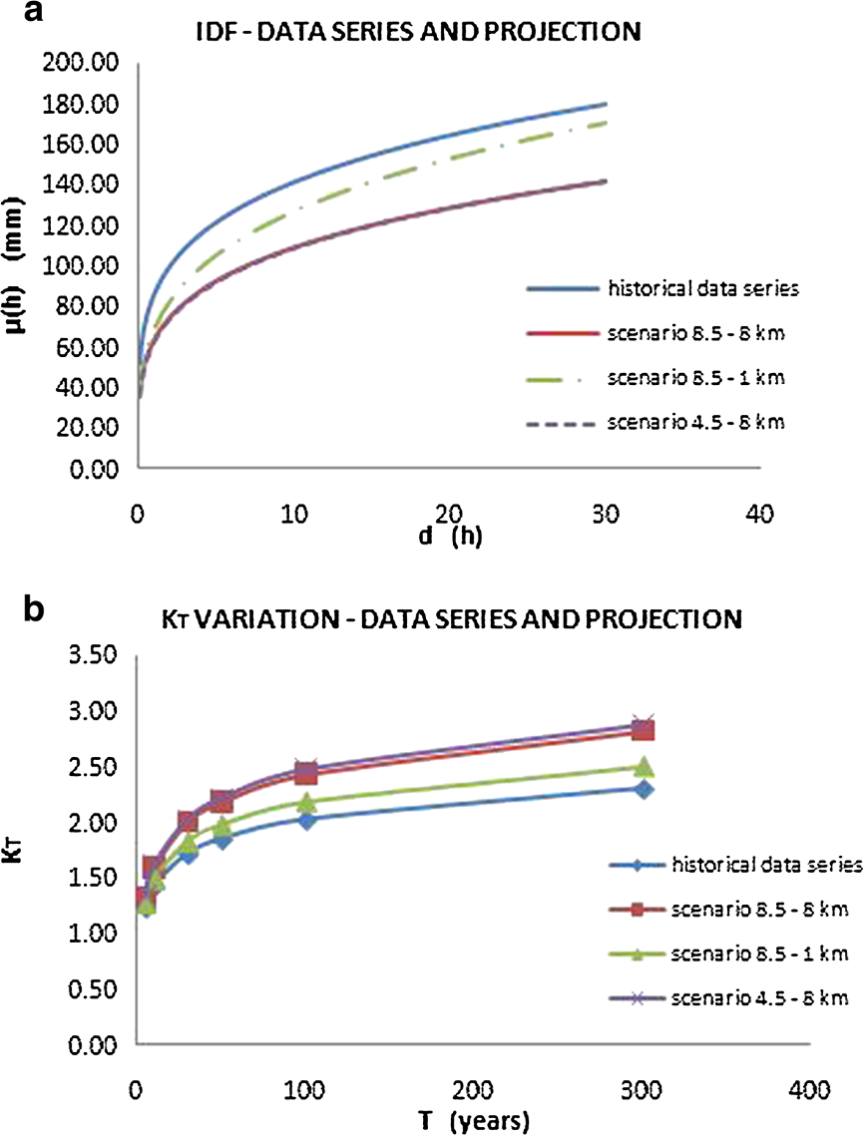
**IDF curves for the city of Douala (a) and variation of growing factor K**_**T**_ **(b) considering both historical data series and climate projections.**

The results, compared with those obtained using only historical data series, are shown in the following Figures 12, 13 and 14, and the obtained parameters are reported in Table 5.

**Table 5:** **IDF parameters for the three test cities**

Addis Ababa				
	Historical data (1964–2010)	CMCC 8.5 8 km	CMCC 8.5 1 km	CMCC 4.5 8 km
a	25.06	20.95	23.97	20.20
n	0.23	0.23	0.253	0.24
KT = 5	1.28	1.32	1.28	1.34
KT = 10	1.50	1.58	1.51	1.61
KT = 30	1.84	1.97	1.86	2.03
KT = 50	2.00	2.15	2.02	2.22
KT = 100	2.21	2.40	2.23	2.47
KT = 300	2.54	2.78	2.56	2.88
**Dar Es Salaam**				
	**Historical data (1958–2010)**	**CMCC 8.5 8 km**	**CMCC 8.5 1 km**	**CMCC 4.5 8 km**
a	36.44	24.97	31.70	26.54
n	0.25	0.29	0.26	0.27
KT = 5	1.23	1.41	1.28	1.37
KT = 10	1.42	1.74	1.50	1.67
KT = 30	1.70	2.24	1.84	2.13
KT = 50	1.83	2.47	2.00	2.34
KT = 100	2.01	2.78	2.21	2.62
KT = 300	2.28	3.26	2.41	3.07
**Douala**				
	**Historical data (1976–2010)**	**CMCC 8.5 8 km**	**CMCC 8.5 1 km**	**CMCC 4.5 8 km**
a	85.17	62.70	67.96	62.44
n	0.22	0.24	0.27	0.24
KT = 5	1.23	1.33	1.27	1.34
KT = 10	1.42	1.59	1.49	1.61
KT = 30	1.71	2.00	1.82	2.02
KT = 50	1.84	2.18	1.97	2.21
KT = 100	2.02	2.43	2.18	2.47
KT = 300	2.30	2.82	2.50	2.87

The IDF curves show that the two different scenarios, considering the same spatial resolution, don’t display substantial deviations. Instead, a meaningful deviation depends on the different downscaling: in fact, as shown, the 1 km downscaling provided projections that afford to capture extreme events.

In terms of frequency, as shown by the curves of growing factor variation K_T_, it’s possible to note how the effect of climate change, in the three test cities, involves a rise of frequency of extreme events. In fact, as shown in Figures 12, 13 and 14(b), keeping constant K_T_, the corresponding return period value is reduced taking in account the climate projections.

Finally, in terms of intensity, the effects of climate change are different for the three cities considered.

For the cities of Dar Es Salaam and Douala, there is a decrease in terms of intensity, in fact the IDF curves that take in account the climate projection are lower than those evaluated with the only historical data series, considering both the downscaling, 8 km and 1 km. Instead, in the case of Addis Ababa, the curve evaluated for the scenario 8.5 referring to a 1 km spatial resolution is very similar to the one evaluated from the historical data.

In order to give useful information and a complete picture of the rainfall pattern of the three test cities, the Probable Maximum Precipitation (PMP) for the three cities was analyzed.

In Tables 6, 7 and 8 the results of the PMP evaluation for the three test cities were shown, using the two different procedures illustrated before. In particular, the k_m_ values have been calculated both with the WMO empirical nomograph and with the (21) equation. The tables show the values evaluated only by historical data and also by historical data and climate projections, in particular referred to the scenario RCP 8.5 with 1 km resolution. Obviously, with increasing duration, the value of PMP increases and, as expected, for Douala the maximum values of PMP occur.

**Table 6:** **Evaluation of k**_**m**_ **and PMP for Addis Ababa**

	1964-2010							1964-2049						
t	***X***_***n***_	σ _n_	X _M_	k _m WMO_	PMP WMO	k _m_	PMP	***X***_***n***_	σ _n_	X _M_	k _m WMO_	PMP WMO	k _m_	PMP
(min)	(mm)	(mm)	(mm)		(mm)		(mm)	(mm)	(mm)	(mm)		(mm)		(mm)
10	18.1	6.3	31.8	10.9	87.5	2.3	32.7	16.0	6.7	31.8	11.8	95.0	2.3	31.5
30	30.4	9.7	53.3	9.6	123.8	2.5	55.0	25.4	10.3	53.3	10.8	135.9	2.7	53.2
60	36.4	11.3	63.3	12.0	171.7	2.6	65.4	31.0	11.8	63.3	13.0	183.9	2.7	62.8
180	38.2	12.3	70.7	13.3	201.5	2.9	73.9	34.2	12.3	70.7	13.9	204.9	2.9	69.9
360	43.7	14.2	83.7	15.4	262.1	3.1	88.2	40.5	13.3	83.7	15.7	249.2	3.2	82.7
720	50.0	16.5	99.2	15.9	312.1	3.4	105.4	47.4	15.3	99.2	16.1	292.5	3.3	97.6
1440	52.5	18.1	110.0	17.6	370.5	3.6	118.4	54.4	20.0	120.3	17.5	404.2	3.2	117.5

**Table 7:** **Evaluation of k**_**m**_ **and PMP for Dar Es Salaam**

	1958-2010							1958-2050						
t	***X***_***n***_	σ _n_	X _M_	k _m WMO_	PMP WMO	k _m_	PMP	***X***_***n***_	σ _n_	X _M_	k _m WMO_	PMP WMO	k _m_	PMP
(min)	(mm)	(mm)	(mm)		(mm)		(mm)	(mm)	(mm)	(mm)		(mm)		(mm)
10	21.6	7.7	42.2	9.6	96.4	3.2	46.6	16.2	8.9	42.2	11.66	120.9	3.3	46.0
30	32.2	11.2	62.4	9.2	135.4	3.8	75.4	23.6	13.4	62.4	11.21	174.1	3.7	73.5
60	39.8	13.1	72.0	11.4	188.8	2.8	77.5	28.8	16.4	72.0	13.41	249.2	2.9	76.8
180	45.4	13.8	74.4	12.2	215.3	3.3	91.9	33.1	18.0	74.4	14.08	286.6	3.2	90.3
360	56.7	17.8	104.4	14.1	309.8	3.2	114.3	41.7	22.6	104.4	15.59	394.2	3.1	112.8
720	69.4	20.3	119.1	14.5	364.6	3.4	140.2	51.5	26.5	119.1	15.75	470.2	3.2	137.5
1440	78.7	23.6	137.0	16.4	466.4	3.3	156.6	60.2	29.9	137.0	17.23	576.4	3.1	154.0

**Table 8:** **Evaluation of k**_**m**_ **and PMP for Douala**

	1976-2010							1976-2050						
t	***X***_***n***_	σ _n_	X _M_	k _m WMO_	PMP WMO	k _m_	PMP	***X***_***n***_	σ _n_	X _M_	k _m WMO_	PMP WMO	k _m_	PMP
(min)	(mm)	(mm)	(mm)		(mm)		(mm)	(mm)	(mm)	(mm)		(mm)		(mm)
10	48.9	21.0	104.4	5.4	162.9	3.0	111.2	38.2	17.9	104.4	5.9	144.4	4.1	112.1
30	79.3	23.8	136.2	5.9	219.7	2.5	139.1	55.2	28.3	136.2	7.1	256.7	3.1	141.7
60	98.5	28.5	192.2	6.1	271.9	4.1	214.4	68.8	34.6	192.2	7.3	322.5	4.0	205.5
180	108.4	34.2	210.0	6.4	325.6	3.5	228.7	76.7	38.7	210.0	8.6	410.7	3.8	223.3
360	126.7	39.5	246.0	9.0	480.7	3.6	268.9	94.3	42.2	246.0	11.1	563.2	4.0	262.6
720	148.2	45.7	288.2	10.7	635.6	3.7	315.9	117.0	45.8	288.2	11.9	661.5	4.2	308.6
1440	161.6	53.1	320.0	13.2	863.2	3.5	349.7	134.5	49.6	320.0	14.2	840.1	4.2	342.2

Moreover, the return period associated with the 24 hours PMP values have been evaluated. As shown in Table 9, the k_m_ value ranges between 13 and 18, using the WMO nomograph, and between 2 and 4, using the (21) equation. The PMP, related to the WMO k_m,_ corresponds to very high values of return period, while, using the k_m_ calculated with the (21), the return period ranges are between 100 and 250 years.

**Table 9:** **Evaluation of return period for the PMP values for the duration of 24 hours**

		Historical data			Historical data + projections		
		k _m_	PMP	T	k _m_	PMP	T
**Addis Ababa**	*WMO*	17.57	370.48	1.E + 05	17.48	404.21	2.E + 05
	*Evaluated*	3.64	118.39	126	3.15	117.47	101
**Dar Es Salaam**	*WMO*	16.44	466.48	1.E + 05	17.23	576.38	1.E + 06
	*Evaluated*	3.30	156.63	157	3.13	154.00	237
**Douala**	*WMO*	13.22	863.15	4.E + 04	14.21	840.09	2.E + 04
	*Evaluated*	3.54	349.67	112	4.18	342.21	99

This result is very interesting and should be pointed out that the stations used to create the WMO nomograph are located mostly in USA, so in an area characterized by rainfall patterns very different from the african one.

So, it is more advisable to use the Hershfield’s procedure to evaluate the PMP that can be used for the design of hydraulic structures in African cities.

Finally, in order to verify the goodness of the applied procedure, a comparison was made with the IDF curve obtained for the station of Bouake (Côte d’Ivoire) in the work of Soro et al. (2010). Indeed, in this work, the curve was evaluated based on rainfall series of durations ranging from 15 minutes to 4 hours.

**Figure 15:**
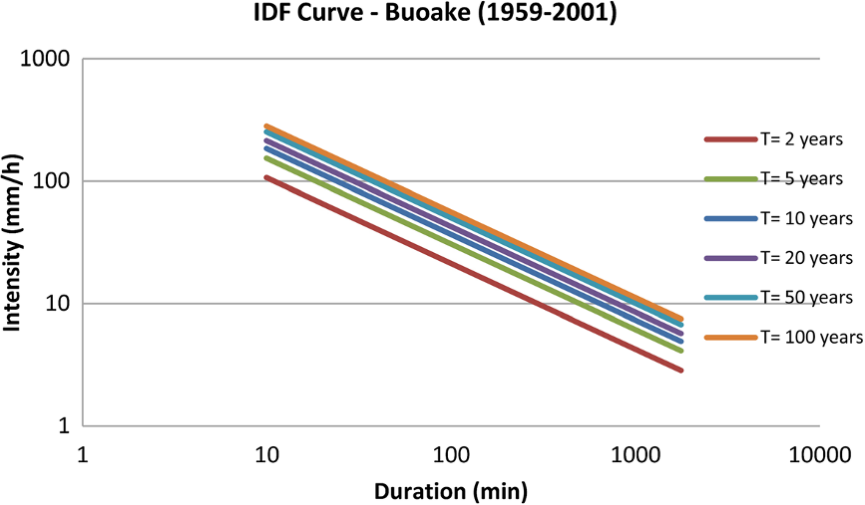
**I DF curves for the city of Bouake (Côte d’Ivoire).**

The curve in Figure 15, on double logarithmic axes, was evaluated using the daily rainfall data, over the period 1959 – 2001, and applying the disaggregation procedure, for different return periods T. The parameters obtained are shown in Table 10. In particular, using the disaggregation procedure, the obtained IDF curve underestimates values less than one hour while overestimates values greater than one hour, although overall the curve is similar to that developed by Soro et al.. More in details, taking in account the values referring, for example, to T =100 years, for a duration less than 2 hours and half, the obtained IDF gives values less than the one obtained from the Soro one, with an average deviation of about 1.6.

**Table 10:** **IDF parameters for Bouake (Côte d’Ivoire)**

Bouake - 1959-2001	
a	36.165
n	0.28
K_T_ = 2	0.92
K_T_ = 5	1.35
K_T_ = 10	1.63
K_T_ = 20	1.91
K_T_ = 50	2.26
K_T_ = 100	2.53

### Conclusive remarks

The present work shows a methodology for the evaluation of the IDF curves from daily rainfall data. In particular, to obtain durations shorter than 24 hours, two different models of disaggregation were applied to the historical data available for the three cities considered, Addis Ababa, Dar Es Salaam and Douala. The IDF curves were obtained later using the probability distribution of Gumbel.

In order to estimate the contingent influence of climate change on the IDF curves, the illustrated procedure was applied to the rainfall projections over the time period 2010–2050 provided by CMCC, for two different emission scenarios and different spatial resolutions (8 km and 1 km).

The analysis of the IDF curves showed moderate deviations between the two scenarios, RCP 4.5 and RCP 8.5, while substantial variations depend on the different downscaling and, in particular, the 1 km downscaling provided projections that afford to capture extreme events.

Analyzing the growing factor K_T_, it is possible to note that the effect of climate change in the three test cities involves a rise of frequency of extreme events. The effects of climate change in terms of intensity are different. In fact, while Dar Es Salaam and Douala, there is a decrease in terms of intensity, considering both the downscaling, 8 km and 1 km, for Addis Ababa, the IDF curve evaluated for the scenario 8.5 referring to 1 km spatial resolution, is very similar to the one calculated by the historical data.

In conclusion, the results of the climate model projections suggest that future rainfall intensity could be subjected to decreases or increases depending on the different area considered, but with an increase in terms of frequency.

Moreover, two different approaches were applied satisfactorily to obtain the PMP in the three test cities for several durations ranging from 10 min to 24 h, using not only historical data but also climate projections (scenario RCP 8.5), In particular, has been pointed out that the PMPs evaluated using the WMO nomograph returns values of return period too high, so the Hershfield’s procedure is the more advisable in order to evaluate the PMP that can be used for design of hydraulic structures in African cities.
